# Metabolomic insights in advanced cardiomyopathy of chronic chagasic and idiopathic patients that underwent heart transplant

**DOI:** 10.1038/s41598-024-53875-7

**Published:** 2024-04-29

**Authors:** Raphaela M. de Oliveira, Mariana U. B. Paiva, Carolina R. C. Picossi, Diego V. N. Paiva, Carlos A. O. Ricart, Francisco J. Ruperez, Coral Barbas, Fernando A. Atik, Aline M. A. Martins

**Affiliations:** 1 School of Medicine, University of Brasilia, Brasilia, Brazil; 2 Laboratory of Protein Chemistry and Biochemistry, University of Brasilia, Brasilia, Brazil; 3 Center of Excellence in Metabolomics and Bioanalysis, University of San Pablo CEU, Madrid, Spain; 4 Institute of Cardiology and Transplantation of the Federal District, Brasilia, Brazil

**Keywords:** Metabolomics, Cardiology, Translational research, Mass spectrometry

## Abstract

Heart failure (HF) studies typically focus on ischemic and idiopathic heart diseases. Chronic chagasic cardiomyopathy (CCC) is a progressive degenerative inflammatory condition highly prevalent in Latin America that leads to a disturbance of cardiac conduction system. Despite its clinical and epidemiological importance, CCC molecular pathogenesis is poorly understood. Here we characterize and discriminate the plasma metabolomic profile of 15 patients with advanced HF referred for heart transplantation – 8 patients with CCC and 7 with idiopathic dilated cardiomyopathy (IDC) – using gas chromatography/quadrupole time-of-flight mass spectrometry. Compared to the 12 heart donor individuals, also included to represent the control (CTRL) scenario, patients with advanced HF exhibited a metabolic imbalance with 21 discriminating metabolites, mostly indicative of accumulation of fatty acids, amino acids and important components of the tricarboxylic acid (TCA) cycle. CCC vs*.* IDC analyses revealed a metabolic disparity between conditions, with 12 CCC distinctive metabolites vs*.* 11 IDC representative metabolites. Disturbances were mainly related to amino acid metabolism profile. Although mitochondrial dysfunction and loss of metabolic flexibility may be a central mechanistic event in advanced HF, metabolic imbalance differs between CCC and IDC populations, possibly explaining the dissimilar clinical course of Chagas’ patients.

## Introduction

Heart failure (HF) is a clinical syndrome associated with alterations in cardiac energy metabolism, such as imbalanced anabolic-catabolic signaling and defects in energy production^1^, and significant phenotypic heterogeneity. One of the predisposing causes is non-ischemic dilated cardiomyopathy (DCM), which usually starts with a long preclinical phase that, regardless of etiology, progresses to hypertrophic remodeling and dilation and systolic dysfunction of the left or both ventricles^2^. Attributed to genetic or non-genetic causes, as in idiopathic dilated cardiomyopathy (IDC) or myocarditis, the molecular spectrum common to DCM subtypes includes a stimulation of the sympathetic adrenergic and renin–angiotensin–aldosterone (RAAS) systems and mobilization of natriuretic peptides to increase vascular tone, inotropism and sodium and water retention^3^.

Among non-ischemic DCM etiologies, chronic chagasic cardiomyopathy (CCC) is highly prevalent in Latin America^4^. As a progressive degenerative inflammatory condition that primarily causes collagen accumulation in myocardial interstitium and disturbance of cardiac conduction system^5^, cardiac muscle involvement in CCC is complex. Heart remodeling occurs as a result of the synergistic effect between immunological and inflammatory factors, cardiac dysautonomia and microvascular disturbances^5^. A recent study also found that cardiomyocytes isolated from Chagas’ patients have higher levels of intracellular inositol 1, 4, 5 trisphosphate (IP_3_), a metabolite that stimulates a significantly increase in diastolic calcium ions (Ca^2+^) concentration and consequently aggravates the contractile disorder^6^.

In this context, metabolic fingerprints have great potential to improve the understanding and molecular signature of chagasic HF previously investigated by other omics^7–9^. However, most reports focus on ischemic and idiopathic heart diseases, excluding patients with CCC despite their clinical and epidemiological importance. Thus, the aim of our study was to characterize and discriminate possible molecular mechanistic events that drives the phenotypical result of non-ischemic HF and CCC patients using a metabolomics approach.

## Results

### Advanced HF vs. control (CTRL)

**Figure 1:**
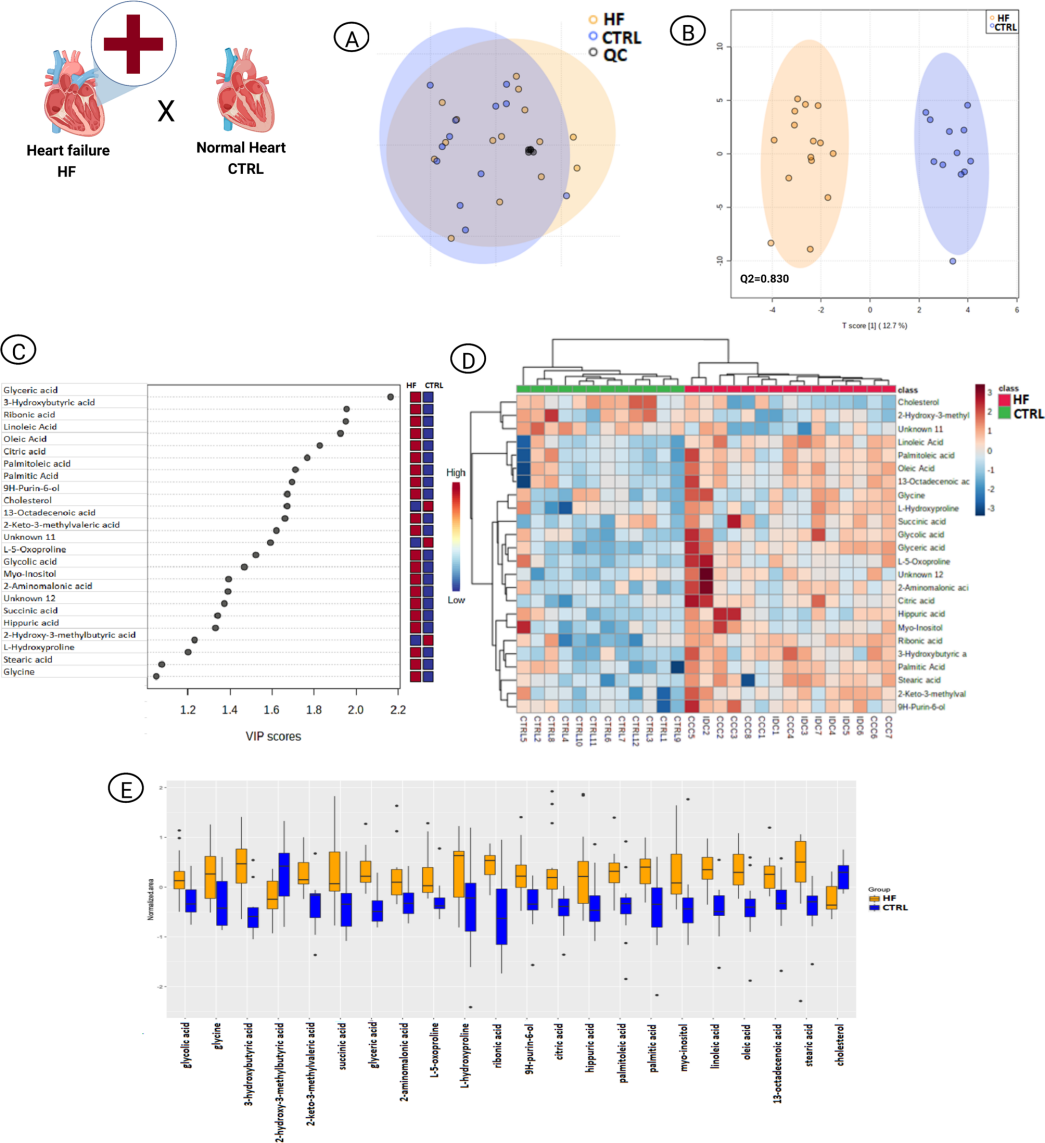
Multivariate analysis of advanced HF^(CCC+IDC)^ and CTRL patients. (**A**) PCA scores plot to evaluate analytical (QC) reproducibility. (**B**) OPLS-DA scores plot (95% confidence; p < 0.05). Each point corresponds to a patient’s sample. (**C**) OPLS-DA VIP scores. The colored boxes on the right indicate the relative intensities of the metabolites in each phenotypic group. (**D**) Heatmap hierarchical clustering of the OPLS-DA discriminant metabolites (VIP score ≥ 1.0). Arrays express samples or metabolites relationship. (**E**) Whiskers box plot of OPLS-DA discriminant metabolites (VIP score ≥ 1.0). The diagram shows the compounds distribution in quartiles for each condition and each point outside the range corresponds to an outlier sample. *CTRL* control, *HF* heart failure, *OPLS*-*DA* orthogonal partial least square discriminant analysis, *PCA* principal component analysis, *QC* quality control, *VIP* variable importance in projection.

**Figure 2:**
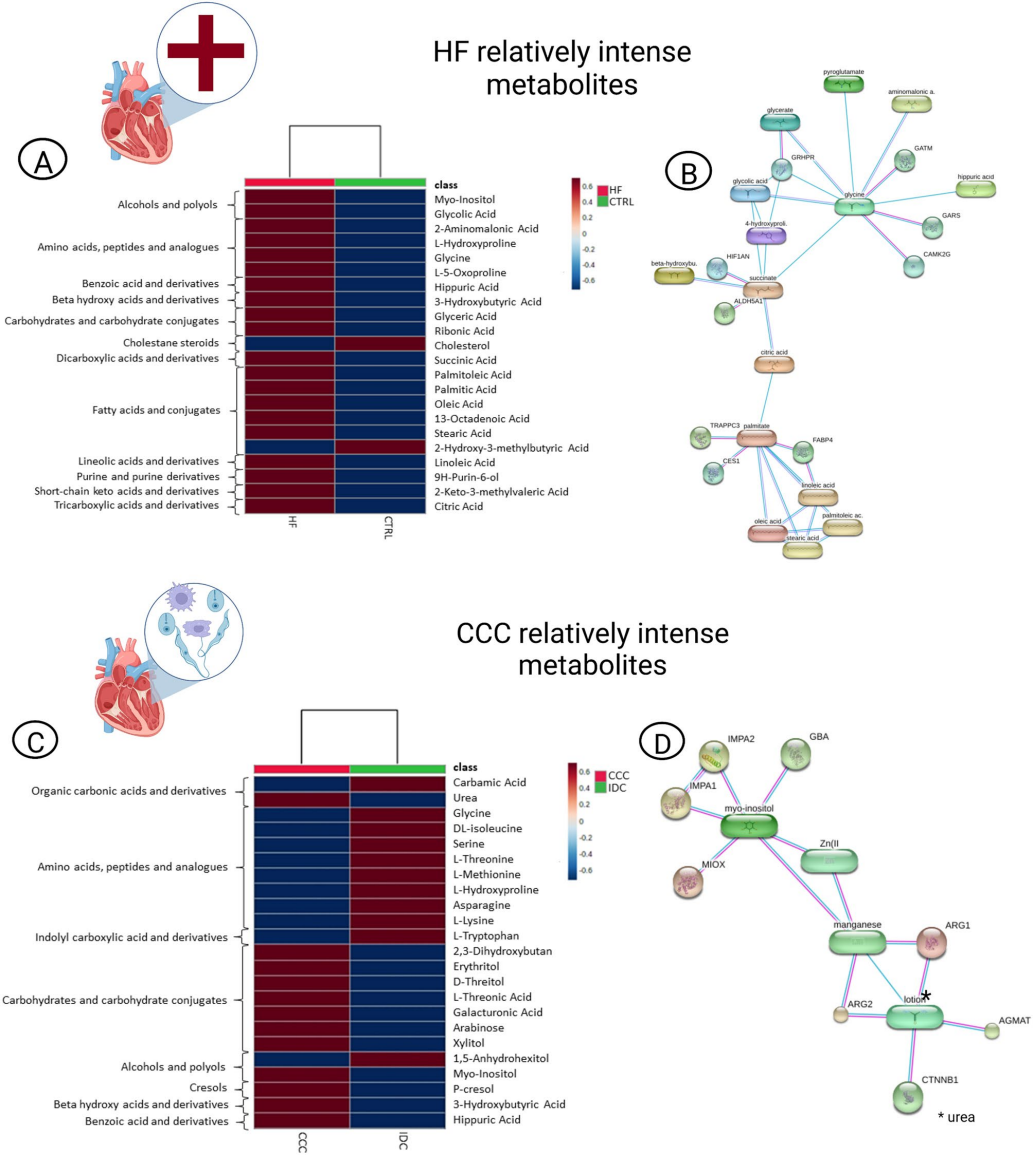
Metabolite network associated with advanced HF^(CCC+IDC)^ and CCC. (**A**) Heatmap class clustering of HF-discriminating metabolites in OPLS-DA (VIP score ≥ 1.0, HF vs. CTRL). (**B**) HF-discriminating metabolites network. (**C**) Heatmap class clustering of CCC-discriminating metabolites in OPLS-DA (VIP score ≥ 1.0, CCC vs*.* IDC). (**D**) CCC-discriminating metabolites network. Spherical nodes represent predicted functional partners and line colors indicate the type of interaction evidence, meaning blue for curated databases, pink for experimentally determined and purple for protein homology. *CCC* chronic chagasic cardiomyopathy, *CTRL* control, *HF* heart failure, *OPLS*-*DA* orthogonal partial least square discriminant analysis, *VIP* variable importance in projection.

The baseline demographic and clinical characteristics of patients with advanced HF are shown in Table 1. With exception of age (p = 0.03), diastolic blood pressure (p = 0.042) and heart rate (p = 0.02), no significant differences were found between CCC and IDC patients. In multivariate statistics, the analytical reproducibility was confirmed by quality control (QC) samples clustering in principal component analysis (PCA) (Fig. 1A). Orthogonal partial least squares-discriminant analysis (OPLS-DA) revealed a differential metabolic profile in cardiac compromise, with Q2 = 0.83 (Fig. 1B) and 21 HF discriminating metabolites (Fig. 1C). Differences in intensity and distribution of these compounds were demonstrated by heatmap (Fig. 1D) and box plot (Fig. 1E). Most of them belong to the class of fatty acids and conjugates or amino acids, peptides and analogues (Fig. 2A). Their interaction network indicated an interactome pathway potentially implicated in the pathophysiology of advanced non-ischemic HF (Fig. 2B). OPLS-DA results for each pathological scenario vs. CTRL subjects are shown in Supplemental Fig. 1 and the univariate findings in Supplemental Table 1.

**Table 1:** Baseline characteristics of CCC and IDC patients.

Characteristics	CCC (n = 8)	IDC (n = 7)	p-value
Male sex	6 (75%)	4 (57.14%)	0.855
Age, y	56.6 (± 10.6)	29.3 (± 21.9)	0.03
Weight, kg	57.5 (± 12.5)	61.1 (± 20.9)	1
BMI, kg/m^2^	21.2 (± 4.81)	22.7 (± 7.93)	1
NYHA functional class^a^	–	–	0.232
I/II	0(0%)	1 (14.29%)	–
III	4(50%)	2 (28.57%)	–
IV	4(50%)	4 (57.14%)	–
Hemodynamic profile^b^	–	–	0.935
A	1 (12.5%)	1 (14.29%)	–
B	4 (50%)	4 (57.14%)	–
C	3 (37.5%)	2 (28.57%)	–
History	–	–	–
Hypertension	1 (12.5%)	1 (14.29%)	1
Dyslipidemia	4 (50%)	1 (14.29%)	0.36
Myocardial infarction	0 (0%)	0 (0%)	–
Stroke	3 (37.5%)	1 (14.29%)	0.506
Current smoking	0 (0%)	1 (14.29%)	1
Atrial fibrillation	2 (25%)	2 (28.57%)	0.506
Physical examination	–	–	–
Systolic BP, mmHg	95.4 (± 16.8)	105 (± 12.8)	0.152
Diastolic BP, mmHg	58.4 (± 9.2)	71.9 (± 13)	0.042
Heart rate, beats/min	64.2 (± 11)	83.1 (± 18.6)	0.02
O_2_ saturation, %	99 (± 1)	99 (± 0.9)	0.7
Echocardiography	–	–	–
LVEF, %	26 (± 7)	25 (± 8)	0.955
LVDD, mm	69.4(± 7.76)	64 (± 9.19)	0.473
Diastolic dysfunction	6 (75%)	7 (100%)	0.344
Right ventricular dysfunction	8 (100%)	7 (100%)	-
Laboratory	–	–	–
Hemoglobin, g/dL	12.7 (± 2.29)	12.1 (± 2.11)	0.601
Urea, mg/dL	80.5 (± 29.9)	64.4 (± 51.9)	0.281
Creatinine, mg/dL	2.2 (± 2.14)	1.21 (± 0.815)	0.129
Sodium, mEq/L	138 (± 1.85)	137 (± 1.99)	1
AST, units/L	35.6 (± 28.1)	18.7 (± 5.38)	0.145
ALT, units/L	30.3 (± 26.5)	17 (± 8.49)	0.442
Bilirubin, mg, dL	1.0 (± 0.92)	1.21 (± 0.85)	0.52
Alkaline phosphatase, units/L	106 (± 43.9)	76 (± 8.28)	0.127
γ-GT, units	97.6 (± 68.7)	98.7 (± 62.8)	0.897
Drugs	–	–	–
ACEI	2 (25%)	1 (14.29%)	0.4
ARB	2 (25%)	3 (42.86%)	0.175
β-blocker	8 (100%)	4 (57.16%)	0.155
Spironolactone	6 (75%)	7 (100%)	0.509
Loop diuretic	6 (75%)	6 (85.71%)	1
Thiazide diuretic	1 (12.5%)	1 (14.29%)	1
Hydralazine/nitrate	4 (50%)	1 (14.29%)	0.36
Digoxin	2 (25%)	0 (0%)	0.509
Amiodarone	5 (62.5%)	1 (14.29%)	0.17
Rassi score^c^	14.8 (± 2.2)	–	–
Cardiac devices^d^	5 (62.5%)	2 (28.58%)	0.23

Data are expressed as mean (± standard deviation) or as number (%).

*ACEI* angiotensin-converting-enzyme inhibitors, *ALT* alanine amainotransferase, *ARB* angiotensin II receptor blockers, *AST* aspartate transaminase, *BMI* body mass index, *CCC* chronic chagasic cardiomyopathy, *GT* glutamyl transferase, *IDC* idiopathic dilated cardiomyopathy, *LVDD* left ventricular diastolic diameter, *LVEF* left ventricular ejection fraction, *NYHA* New York Heart Association, *O*_2_ oxygen.

^a^Functional classification according to symptom severity.

^b^Stratification by degree of congestion and adequacy of perfusion.

^c^Risk score for predicting death in patients with CCC.

^d^Implantable cardioverter defibrillators, pacemarkers and cardiac resynchronization therapy (biventricular pacing).

### CCC vs. IDC vs. CTRL

**Figure 3:**
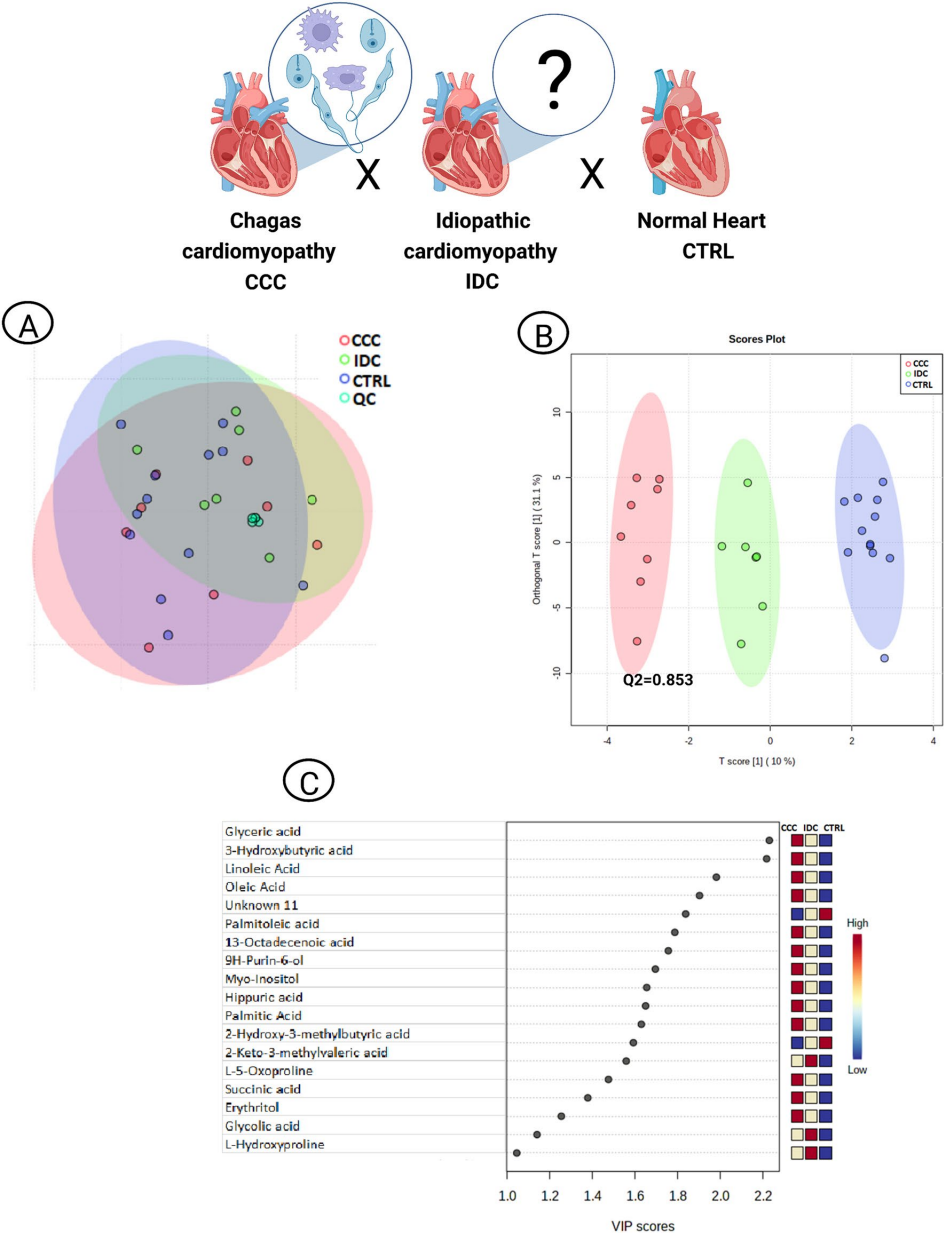
Multivariate analysis of CCC, IDC and CTRL patients. (**A**) PCA scores plot to evaluate analytical (QC) reproducibility. (**B**) OPLS-DA scores plot (95% confidence; p < 0.05). Each point corresponds to a patient’s sample. (**C**) OPLS-DA VIP scores. The colored boxes on the right indicate the relative intensities of the metabolites in each phenotypic group. *CCC* chronic chagasic cardiomyopathy, *CTRL* control, *IDC* idiopathic dilated cardiomyopathy, *OPLS*-*DA* orthogonal partial least square discriminant analysis, PCA principal component analysis, *QC* quality control, *VIP* variable importance in projection.

QC samples clustering attested the consistency of the experimental method in PCA (Fig. 3A). OPLS-DA identified distinct profiles for each condition, with greater distance between CCC and CTRL groups, with Q2 = 0.85 (Fig. 3B). Variations in the relative intensity of 18 metabolites were crucial in characterizing the scenarios (Fig. 3C). The results of univariate analysis are shown in Supplemental Table 2.

### CCC vs. IDC

**Figure 4:**
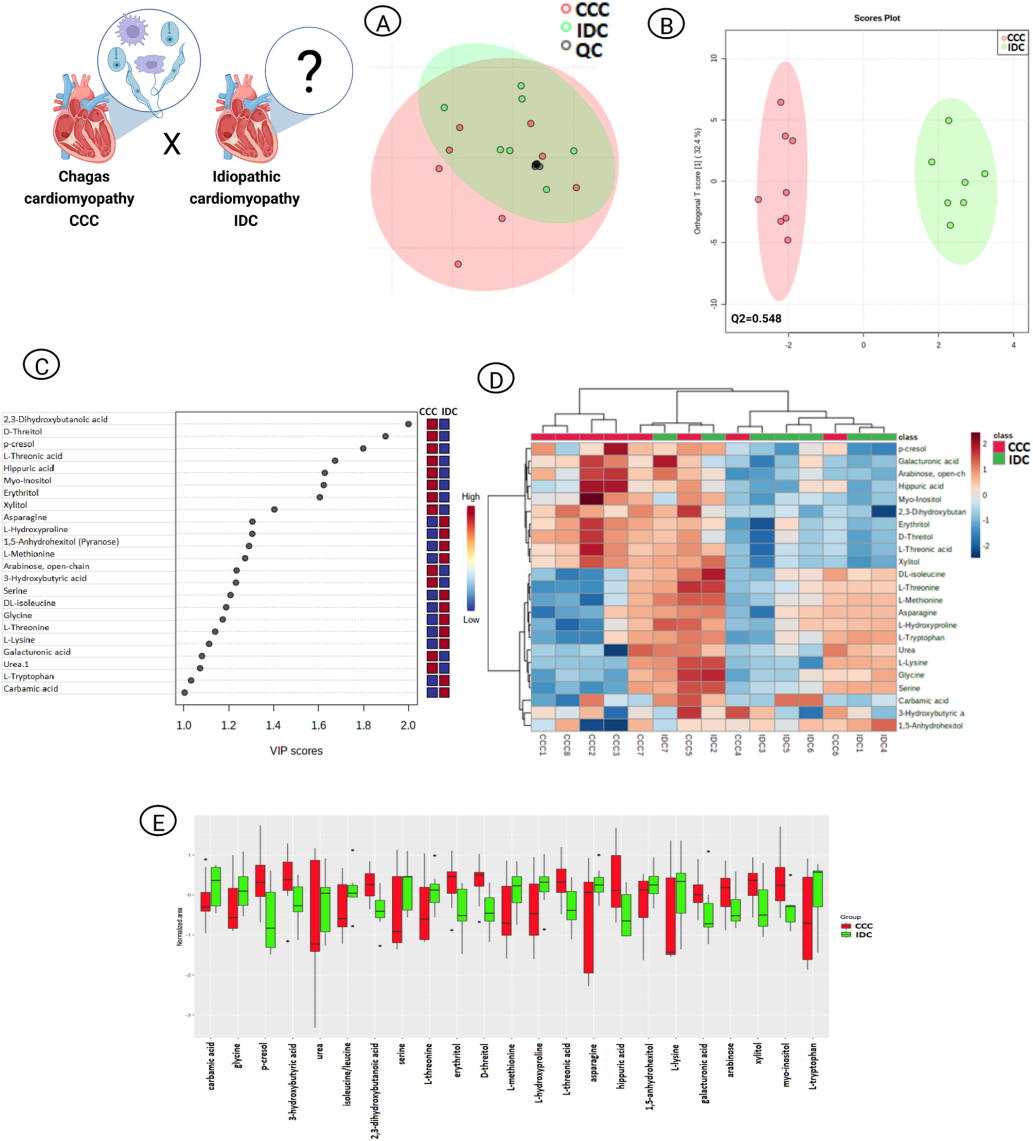
Multivariate analysis of CCC and IDC patients. (**A**) PCA scores plot to evaluate analytical (QC) reproducibility. (**B**) OPLS-DA scores plot (95% confidence; p < 0.05). Each point corresponds to a patient’s sample. (**C**) OPLS-DA VIP scores. The colored boxes on the right indicate the relative intensities of the metabolites in each phenotypic group. (**D**) Heatmap hierarchical clustering of the OPLS-DA discriminant metabolites (VIP score ≥ 1.0). Arrays express samples or metabolites relationship. (**E**) Whiskers box plot of OPLS-DA discriminant metabolites (VIP score ≥ 1.0). The diagram shows the compounds distribution in quartiles for each condition and each point outside the range corresponds to an outlier sample. *CCC* chronic chagasic cardiomyopathy, *IDC* idiopathic dilated cardiomyopathy, *OPLS*-*DA* orthogonal partial least square discriminant analysis, *PCA* principal component analysis, *QC* quality control, *VIP* variable importance in projection.

The method’s reliability was ratified by QC samples clustering in PCA (Fig. 4A). OPLS-DA revealed a metabolic discrimination between the pathological conditions, with Q2 = 0.54 (Fig. 4B) and 12 CCC distinctive metabolites *vs.* 11 IDC representative metabolites (Fig. 4C). Differences in intensity and distribution of these compounds were confirmed by heatmap (Fig. 4D) and box plot (Fig. 4E). The majority of CCC relatively intense metabolites belong to the class of carbohydrates and carbohydrate conjugates, while those important for IDC characterization mostly belong to the class of amino acids, peptides and analogues (Fig. 2C). CCC’s metabolites interaction network suggested a possible pathway particularly associated with the pathophysiology of Chagas Disease (ChD)-related HF (Fig. 2D).

## Discussion

### Metabolite impairment in advanced HF pathophysiology

Although the clinical evolution of patients with ChD-related HF is worse compared to non-Chagas' patients^10^, the molecular features that differentiate the pathogenesis and, consequently, the clinical outcome of CCC and IDC have not yet been elucidated. Here, OPLS-DA results demonstrated that patients with advanced HF, regardless of the cause, exhibit a global metabolic disturbance (Fig. 1B) with higher abundance of 21 identified metabolites (Fig. 1C–E).

Under physiological conditions, the adult heart is metabolically flexible and generates adenosine triphosphate (ATP) from several substrates, such as fatty acids (FAs), lactate, glucose, ketones and amino acids (AAs), obtained continuously from the blood and directed to mitochondrial oxidative phosphorylation (95%) and glycolysis (5%)^11–13^. Up to 60% of mitochondrial ATP production results from FA oxidation, however due to its greater need for oxygen and compromised mitochondrial oxidative capacity, failing hearts tend to increase glucose consumption via anaerobic glycolysis as a compensatory mechanism for maintaining ATP levels^11,13–16^. Metabolic reprogramming in HF also includes increased oxidation of ketone bodies and reduced oxidation of lactate, branched-chain amino acids (BCAA) and glucose, impairing overall ATP production by the tricarboxylic acid (TCA) cycle flux^11,13^.

Indeed, the accumulation of FAs, AAs and important TCA cycle components (citric and succinic acids) observed in plasma of HF patients in the present study (Fig. 2A) suggests an important mitochondrial disfunction and consequent loss of metabolic flexibility, which may contribute to the known reduction of up to 30% of ATP content in failing hearts^11,13,17^. As channeled by HF’s relatively intense metabolites network, these two major pathways may interact along as shown in Fig. 2B.

The effort to sustain energy demand during pathological hypertrophy leads to maladaptive circuits, such as increased mitochondrial protein acetylation, which produce mitochondrial stress and eventually mitochondria-initiated cell death^18^. From a clinical perspective, this metabolic remodeling may be expressed in left ventricle (LV) hypertrophy progression, as it decreases ventricular compliance and consequently increases filling pressure, in addition to constituting an arrhythmogenic substrate by disorganizing tissue cytoarchitecture^19^. Furthermore, free FAs concentration in plasma is independently associated with HF incidence and adverse outcome^20,21^, and lipid moieties accumulation due to extrapolation of mitochondrial oxidative capacity may result in lipotoxicity and lead to myocardial dysfunction^22,23^.

### Metabolite impairment in CCC and IDC pathophysiology

Comparing CCC and IDC individually to CTRL samples, OPLS-DA results indicates a metabolic profile disparity between the pathological settings, with greater discrimination of CCC in relation to the physiological condition (Fig. 3B and C), corroborating the clinical observations on the outcome and prognosis of these patients^24–26^. Other omics studies have also identified differences among HF etiologies. Cunha-Neto et al.^27^ characterizing gene expression profiles of CCC and IDC myocardial tissues, reported that several immune response, lipid metabolism and mitochondrial oxidative phosphorylation genes were specifically up-regulated in CCC, with a prominence for interferon-gamma (IFN-γ)-inducible genes. Teixeira et al.^28^ performing a comparative proteomic study on CCC, IDC and ischemic cardiomyopathy myocardial tissue samples, found that patients with CCC had the lowest expression of several mitochondrial energy metabolism and FA beta-oxidation proteins, and that high levels of IFN-γ in CCC cardiomyocytes reduce mitochondrial transmembrane potential.

The direct confrontation between CCC and IDC scenarios revealed that, although the predictive ability of OPLS-DA was not as good as the other scenarios – a fact that we attribute to the HF phenotype as a common outcome –, patients with a history of ChD display a distinct metabolic signature (Fig. 4B–E), especially in terms of carbohydrate and AA metabolism (Fig. 2C). Although the selection criteria applied were aimed to minimize the confounding factors, the fact that this was a real-life study did not allow us to reduce the influence of other external factors, such as diet and chronical use of medication, that explain the intensities of xylitol/erythritol and galacturonic acid/arabinose, respectively. Here, such findings are qualitative in nature and were beyond the scope of the present study, but it is advisable that these characteristics be considered in future translational studies.

HF hypercatabolic status is also marked by degradation of skeletal muscle proteins to measure up myocardium dependence on AAs to maintain cardiac ATP levels, leading to cachexia^29,30^. In HF advanced stages, the reduction of AA levels can also be explained by the development of pathogenic gut flora, reported in more than three quarters of class II to IV patients. Clinically important, this intestinal impairment may alter protein metabolism by reducing intestinal absorptions of vitamin B12, folic acid, and vitamin K^31^. In addition, myocardium remodeling^32^ and BCAA overconsumption^33^ possibly contribute to low AA levels. Wang et al.^34^ observed that essential AAs plasma levels, except phenylalanine, were lower in patients that had experienced HF-related re-hospitalization or death in comparison to those who did not. Therefore, considering the diverse course of CCC progression compared to other HF etiologies as well, the decrease in the relative intensity of plasma AAs in patients with CCC of the present study may corroborate such indication of disease severity.

More specifically looking at AAs that have been observed as discriminant between the pathological conditions in our work, CCC patients exhibited lower levels of threonine, previously reported by Hennig et al.^35^. Among other metabolic changes, their fingerprinting approach revealed that threonine levels of rat myoblast were completely depleted in all *T. cruzi* infected conditions, treated with different anti-chagasic drugs or untreated. Similar results were found for *T. brucei procyclic* form, in which threonine is the AA most rapidly metabolized by the parasite for lipid biosynthesis^36^.

Recently, Saleem et al.^37^ found that not only plasma levels of threonine were decreased in HF patients compared to CTRL subjects, but methionine, isoleucine, serine and lysine as well. Methionine in particular, was also classified as an independent and significant predictor of HF in their multivariate regression analysis. In our study, these AAs were also reduced in CCC patients compared to IDC, and according to Aquilani et al.^33^ both AAs number and reduced arterial rates are related to HF severity, thus corroborating the distinct outcome in ChD-related HF. Likewise, the authors also highlighted that methionine levels progressively decrease as the disease worsens.

Tryptophan (Trp) levels also differed between the two HF etiologies in our experiments, with lower intensity in CCC patients. Trp is an essential AA particularly important for proliferation of intracellular pathogens, such as *T. cruzi* amastigotes during the chronic phase of ChD. Host cell dependence on Trp leads to a susceptibility to Trp deprivation by indoleamine 2,3 dioxygenase (IDO)-mediated degradation^38^. However, Trp starvation together with the accumulation of its active catabolite products (kynurenines) can inhibit proliferation, or promote T cell anergy and death, and modulate helper T cell response^39–42^. Reinforcing this double-edged sword effect. Marañón et al.^43^ revealed that IDO activity is higher in patients with ChD compared to CTRL subjects and higher in those in symptomatic chronic cardiac or digestive phase than in asymptomatic patients, establishing a correlation between the enzyme activity status and the transition to chronic infection. They also reported that administration of benznidazole, an anti-chagasic medication, decreased IDO activity in symptomatic patients. Thus, this reduced intensity of Trp observed in CCC patients of our study may be a consequence of its catabolism mediated by the ascending enzymatic activity of IDO.

An increase in CCC myo-inositol levels compared to IDC was also evident in our study. Increased expression of IP_3_ receptors (IP_3_Rs) is a general and key mechanism in the remodeling of Ca^2+^ signaling during heart disease, with an arrhythmogenic effect during ventricular hypertrophy^44^. Considering that inositol phosphates (IPs) are phosphorylated derivatives of myo-inositol, the increase in myo-inositol levels could be part of a compensatory response to provide an alternative pathway for mobilizing intracellular Ca^2+^ release in advanced stages of HF, as suggested by Deidda et al.^45^. In compliance, Mijares et al.^6^ showed that IP_3_R activators induced a greater elevation of diastolic Ca^2+^ in Chagas’ cardiomyocytes compared to those of non-chagasic individuals. Furthermore, Chagas’ cardiomyocytes had a reduced sarcoplasmic reticulum Ca^2+^ loading, higher level of intracellular IP_3_, and compromised contractile properties as well, correlating to (New York Heart Association) NYHA classifications.

CCC patients also had increased levels of urea and hippuric acid compared to the IDC group (Fig. 2C and 4C). Together with our complementary investigations on CCC relatively intense metabolites interaction network (Fig. 2D), these results suggest the hypothesis that the urea cycle of detoxification may be unusually overloaded in ChD-related HF, thereby contributing to the severity of the condition as renal dysfunction is considered an independent outcome predictor in HF^46–48^. Although such research is beyond the planned scope of the present study, there is indeed an interrelationship between heart and kidney injuries clinically described as cardiorenal syndrome (CRS), which leads to an accumulation of uremic toxins in the body^49^. Applied to the cardiologist’s practice, CRS is common in HF patients, associated with worse prognosis and secondary to multiple pathophysiological mechanisms, such as hemodynamic changes leading to venous renal congestion^50^. Moreover, unbalanced protein metabolism in association to kidneys impairment may also culminate in uremia, condition in which levels of urea, myo-inositol and hippuric acid are knowingly increased^51,52^.

Still on uremic toxins, our data show that p-cresol levels, which is an end product of protein metabolism, were increased in CCC patients compared to the IDC group. In line with our findings, a growing number of publications have confirmed that renal patients reveled an emerging role for this specific metabolite in cardiovascular disease and mortality^53,54^. In ChD particularly, Gironès et al.^55^ revealed a marked increase of p-cresol in both heart tissue and plasma of *T. cruzi* infected mice, suggesting that alterations in p-cresol metabolism may be associated with increased cardiac stress in acute myocarditis.

**Figure 5:**
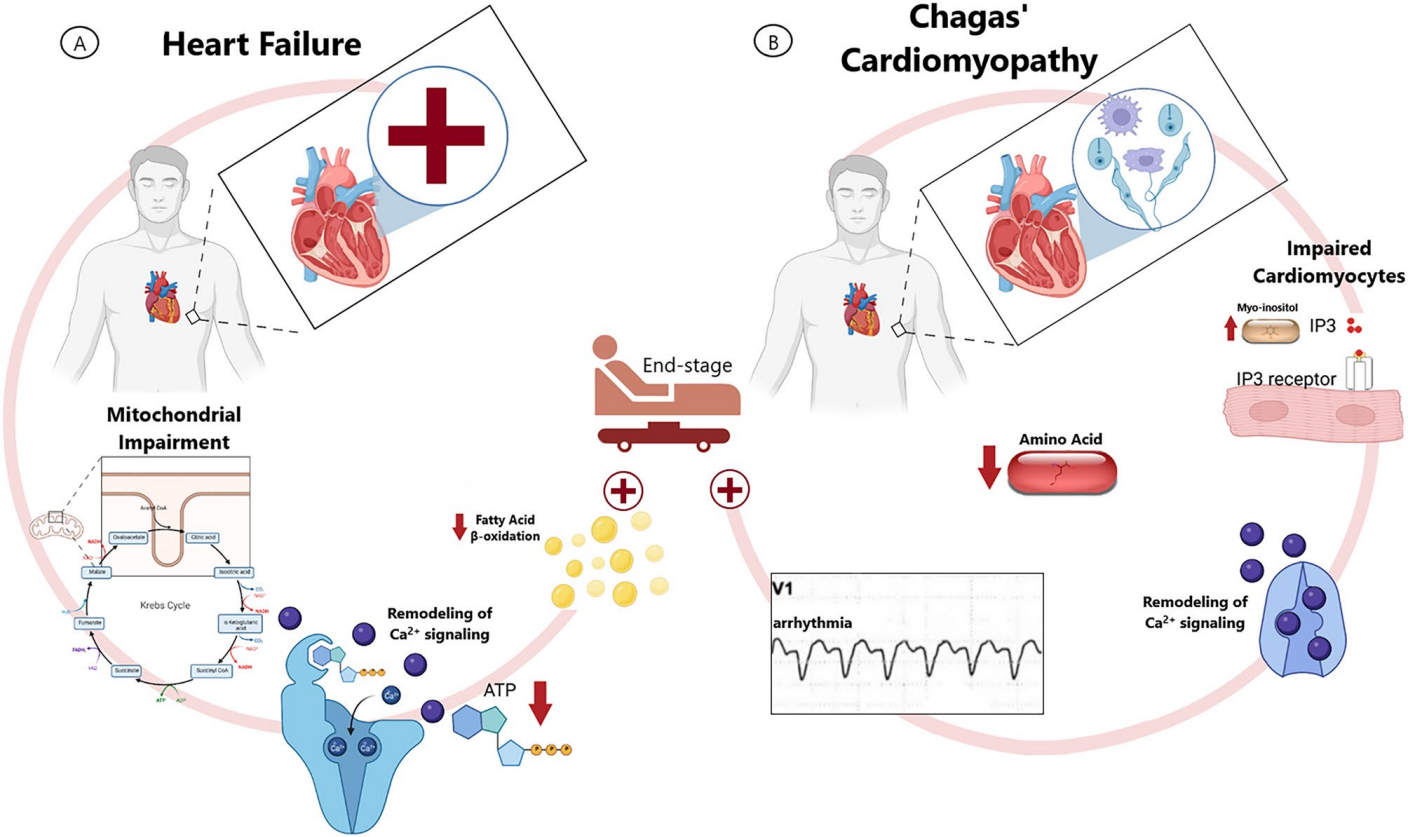
Pathophysiology of advanced HF and CCC from a translational metabolomics basis. (**A**) Link between impaired mitochondrial energy metabolism, FAs and TCA cycle AAs accumulation, LV hypertrophy progression and end-stage HF. (**B**) Link between ChD, impaired cardiomyocytes, higher levels of myo-inositol and intracellular IP_3_, remodeling of Ca^2+^ signaling, arrhythmia and end-stage HF. *AA* amino acid, *Ca*^2+^ calcium, *CCC* chronic chagasic cardiomyopathy, *ChD* chagas’ disease, *CTRL* control, *FA* fatty acid, *HF* heart failure, *IP*_3_ inositol 1,4,5 trisphosphate, *LV* left ventricle, *TCA* tricarboxylic acid.

In summary, our data reveal novel metabolomic insights into HF molecular events that drive the distinct clinical course of CCC patients from those with non-ischemic DCM. Although impairment of mitochondrial oxidative capacity may be a central mechanistic event in predisposing to HF (Fig. 5A), the associated metabolic imbalance differs between CCC and IDC populations especially in terms of AA metabolism. Tryptophan, methionine, isoleucine, serine and lysine had lower abundance in CCC samples compared to IDC. On the other hand, some uremic toxins, such as urea, hippuric acid and p-cresol were more intense in CCC, possibly indicating an overload of the urea detoxification cycle. Myo-inositol, also known to be implicated in the intracellular remodeling of Ca^2+^ signaling and arrhythmias occurrence, was also more intense in ChD-related advanced HF (Fig. 5B).

From a translational perspective, understanding the functionality of these effector-metabolites in CCC may deepen our knowledge on the metabolic changes that significantly influence the cardiomyopathy development, progression and outcome. We recognize that the number of patients in each biological scenario is not ideal, which may have impacted the analysis of clinical data and pairing of participating patients. Nonetheless, the variables analyzed is this pilot work (metabolites) are excellent discriminant in two of the proposed scenarios (Q2 > 0.8). We also acknowledge that a validation of these findings with a larger cohort is needed, in order to translate these important findings into clinics. Future studies should consider including patients with ChD and CCC at various stages to add prognostic value to this matter.

## Patients and methods

### Study population and experimental design

The present investigation was carried out as a prospective pilot study designed in a real-life context to test the adequacy of plasma metabolomic profiling for differentiating advanced non-ischemic and ChD-related HF. Patients enrolled in the specialized HF program at the Institute of Cardiology and Transplantation of the Federal District (ICTDF) in Brasília, Brazil, between July 2017 and July 2019 were recruited. Those with advanced HF secondary to non-ischemic DCM referred for heart transplantation (HT) were eligible after chagasic and idiopathic etiologies confirmation. All patients were in stage D (refractory to clinical treatment)^56^, had HF with reduced ejection fraction (HFrEF), and belonged to NYHA classes III or IV^57^. Those undergoing retransplantation, aged > 70 years, with significant hypertension, history of acute myocardial infarction, or coronary, valvular, cerebrovascular, peripheral vascular, hepatic and severe pulmonary diseases were excluded. In total, 15 patients with advanced HF were enrolled – 8 patients with CCC and 7 with IDC.

Twelve heart donors representing the CTRL scenario were also included to experimentally reproduce physiological conditions and ultimately discriminate the molecular characteristics particularly associated with non-ischemic HF, CCC and IDC advanced stages. To preserve the sample collection method, CTRL enrollment criteria included not only the absence of previous or current heart disease, but also referral for evaluation with the same clinical team responsible for HF patients at HT. Heart donors with positive and confirmatory serological tests for ChD, hepatitis B, hepatitis C, syphilis, toxoplasmosis, cytomegalovirus, HIV and/or HTLV I and II were excluded. The study inclusion and exclusion criteria for both HT recipient and donors were based on the recommendations of the International Society for Heart and Lung Transplantation (ISHLT) guideline^58^, 3rd Brazilian heart transplant directive^59^ and Ordinance MS-GM nº 2.600 (October 21, 2009). The selected number of samples included together with the sensitivity of the analytical technique and the complementary assessment of clinical aspects represent, to a lesser extent, a necessary initial step for a larger translational experiment.

### Ethics statement

This research was approved by the Ethical Review Committee of the ICTDF, Brasilia, Brazil (13158619.7.0000.0026). Written informed consent was obtained from all participants and all methods were performed in accordance with the relevant guidelines and regulations.

### Sample collection and preparation

Peripheral blood samples were collected from the central venous access intraoperatively before heparinization and sternotomy. Following centrifuge-induced fractionation in ethylenediamine tetraacetic acid (EDTA) tubes, 50 μL aliquots of plasma were diluted in 150 μL of cold acetonitrile containing 4-nitrobenzoic acid as internal standard. Supernatants aliquots (100 μL) were lyophilized, reconstituted in 10 μL of o-methoxyamine hydrochloride in pyridine solution and then added 10 µL of O-bistrifluoroacetamide (BSTFA) containing 1% chlorotrimethylsilane (TMCS), for derivatization. 4 blank samples, using 50 µL of Milli-Q® water, and 3 QC samples, consisting of a pool of 100 µL of each plasma, were also prepared randomly. All sample preparation steps are described in detail in the Supporting Information.

### GC–MS/MS analysis

Samples were randomly analyzed by a gas chromatograph (GC 7890A, Agilent Technologies, CA, US) equipped with an automatic liquid sampler (ALS 7693, Agilent Technologies, CA, US) coupled to a quadrupole time-of-flight mass spectrometer (Q-TOF 7200, Agilent Technologies, CA, US). The analysis was performed using a previously developed method^60^ with the analytical conditions described in detail in the Supporting Information.

### Data processing and analysis

After spectral deconvolution in Unknowns Analysis software (Agilent Technologies, CA, US), all GC–MS raw data were checked using MassHunter Qualitative software (version 10.0) to determine the data quality, the system mass accuracy, and the reproducibility of the QC sample, IS injections and data integration. Putative identification was performed using MassHunter Qualitative software (version B.10.00, Agilent Technologies, CA, US), NIST MS Search (Gaithersburg, MD, US), an in-house library (PCDL) and the Fiehn 2013 and NIST17 databases. Univariate analyzes were conducted using the R statistical language (version 4.1.0) following the packages Nortest, Stats, Onewaytests and Ggplot2^61,62^. Data normality was assessed by Shapiro–Wilk’s test and those with parametric distribution were tested for heteroscedasticity (F test) and by an appropriate analysis of variance (ANOVA). Non-parametric data were compared by Kruskal–Wallis test. Tukey’s and Dunn’s post hoc tests, for parametric and non-parametric data, respectively, examined the differences between all paired combinations of groups. Multivariate statistics were conducted in parallel in MetaboAnalyst 4.0^63^ using the same normalized data matrix. Metabolites of greater contribution to OPLS-DA scenarios were also evaluated by STITCH^64^ targeting the highest confidence score (0.900) in neighborhood. All the data processing steps are described in detail in the Supporting Information.

### Supplementary Information

Supplementary Information.

## Data Availability

The dataset generated for this study can be found in the MassIVE repository under the link https://massive.ucsd.edu/ProteoSAFe/dataset.jsp?task=b30335722ed249afa57e600941f560aa.
