# Supplementary material for: Metabolomic insights in advanced cardiomyopathy of chronic chagasic and idiopathic patients that underwent heart transplant
Source: Sci Rep. 2024 Apr 29;14:9810. doi: 10.1038/s41598-024-53875-7 (PMC11059181; doi:10.1038/s41598-024-53875-7)
Supplement: Supplementary file 1 — Supplementary Information. [file 41598_2024_53875_MOESM1_ESM.docx]

Supplementary Material

Metabolomic Insights in Advanced Cardiomyopathy of Chronic Chagasic and Idiopathic Patients that Underwent Heart Transplant

Raphaela M. de Oliveira1^†^, Mariana U. B. Paiva^†^, Carolina R. C. Picossi^†^, Diego V. N. Paiva, Carlos A. O. Ricart, Francisco J. Ruperez, Coral Barbas, Fernando A. Atik*, Aline M. A. Martins*

*** Correspondence:** Corresponding Authors: atikf@me.com; alin3.m4rtins@gmail.com

# Supplementary Methods

## Sample Preparation

50μL aliquots of EDTA plasma obtained from peripheral blood were diluted in 150μL of cold acetonitrile containing 15ppm of internal standard for derivatization (4-nitrobenzoic acid) and vigorously vortexed for 2min. Supernatants aliquots (100μL) were lyophilized and then reconstituted in 10μL of o-methoxyamine hydrochloride (15ppm) in pyridine solution, vigorously vortexed for 2min and homogenized for 3 cycles sonication (10sec in step, 40% intensity). Samples were kept in the dark at room temperature for 16h. In the next day, 10µL of N, O-bistrifluoroacetamide (BSTFA) containing 1% chlorotrimethylsilane (TMCS) were added to each vial and incubated (at 70ºC/1h, then in dark, room temperature/1h). 100µL of 20ppm of tricosane (internal standard for injection) were added to each vial, vortexed and centrifuged (2500rpm/15min/20ºC). 4 blank samples, using 50µL of Milli-Q® water, and 3 quality control (QC) samples, consisting of a pool of 100µL of each plasma, were also prepared randomly.

## GC-MS/MS Analysis

1μL of derivatized sample was loaded in split mode at 1:10 ratio onto a J&W 122-5532G pre-column of 10m (Agilent Technologies, USA) follow by a DB5-MS (95% dimethyl/5% diphenylpolysiloxane film) analytical column (30m x 0.25mm x 0.25µm). Elution temperature gradient was 60ºC to 325°C at a rate of 10°C/min, keeping at 325°C for 10min. The injection port was held at 250°C and gas stream flow (helium) adjusted to 1ml/min. Mass spectra were obtained in 50-500 m/z scan mode with the electron ionization source at -70 eV and 5 spectra/second acquisition. A mixture of n-alkanes (C_8_-C_28_) analyzed at the beginning of the experiment assisted in the acquisition and QC samples were strategically distributed to check the stability of the system. All chromatograms and internal standards peaks were carefully analyzed to check compounds derivatization quality and analytical stability.

## Data Processing and Analysis

After spectral deconvolution in Unknowns Analysis software (Agilent Technologies) and peak alignment in Mass Profile Professional software (MPP, version 14.9), a calibration file was built (retention time and index of the n-alkanes). The compounds areas were integrated using Mass Hunter Quantitative software (version B.08.00, Agilent Technologies) with one ion as the quantifier and two ions as the qualifiers. Peak integration was manually checked and corrected, and a table containing the identified analytes and/or molecular entities with their respective areas in each sample (minus the mean area of the blanks) was generated. This data was normalized by internal standard (4-nitrobenzoic acid) and analytes with a QC variation ≥ 30% were removed.

# Supplementary Figures and Tables

## Supplementary Figures

**
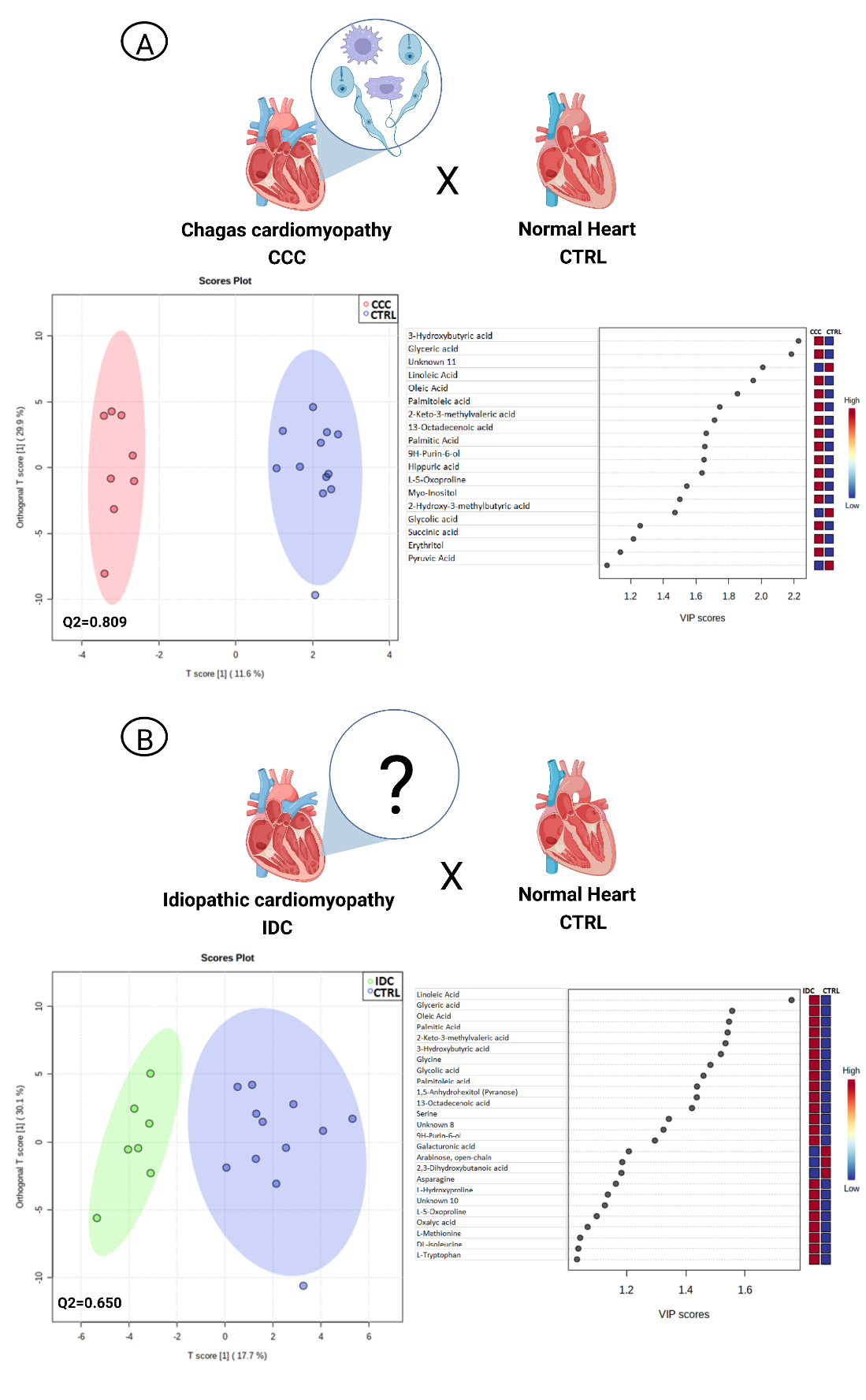
**

Supplementary Figure 1. OPLS-DA of pathological and control patients. **(A)** Scores plot and VIP scores of CCC *vs.* CTRL. **(B)** Scores plot and VIP scores of IDC *vs.* CTRL. The elliptical regions correspond to the 95% confidence interval (p <0.05) and each point in the scores plot corresponds to a sample. The colored boxes on the right indicate the relative intensities of the metabolites in each phenotypic group and warmer colors represent more intense distribution. CCC – chronic chagasic cardiomyopathy; CTRL – control; IDC – idiopathic dilated cardiomyopathy; OPLS-DA – orthogonal partial least square discriminant analysis; VIP – variable importance in projection.

## Supplementary Tables

Supplementary Table 1. Statistically significant metabolites between chronic HF and CTRL patients.

| **Compounds** | **FDR** | **QC (RSD%)** | **Variation (%)** |
| --- | --- | --- | --- |
| Glyceric acid | 0.0002 | 18.28 | 166.9 |
| Linoleic acid | 0.001 | 5.44 | 174.1 |
| L-5-Oxoproline | 0.005 | 18.82 | 76.0 |
| 3-Hydroxybutyric acid | 0.005 | 16.16 | 400.1 |
| Oleic acid | 0.005 | 5.47 | 145.2 |
| 2-Keto-3-methylvaleric acid | 0.006 | 10.30 | 89.3 |
| Cholesterol | 0.006 | 9.56 | -35.9 |
| Palmitic Acid | 0.01 | 4.47 | 160.6 |
| 13-Octadecenoic acid | 0.01 | 5.68 | 87.4 |
| Myo-Inositol | 0.02 | 18.50 | 28.7 |
| Palmitoleic acid | 0.02 | 5.24 | 121.5 |
| Glycolic acid | 0.02 | 19.80 | 79.0 |
| Unknown 11 | 0.02 | 15.76 | -64.6 |

FDR – false discovery rate; QC – quality control; RSD – relative standard deviation.

Supplementary Table 2. Statistically significant metabolites between CCC, IDC and CTRL patients. The results of each compound are presented for total and partial comparisons. None of the metabolites match the post hoc criteria in the CCC *vs.* IDC test.

| **Compounds** | **Post hoc p-value** | **Post hoc p-value**  **CCCxCTRL** | **Post hoc p-value**  **IDCxCTRL** | **QC (RSD%)** | **Variation (%) CCCxCTRL** | **Variation (%) CTRLxIDC** |
| --- | --- | --- | --- | --- | --- | --- |
| Glyceric Acid | 0.0003 | 0.002 | 0.003 | 18.3 | 186.1 | -59.2 |
| Linoleic Acid | 0.0015 | 0.005 | 0.008 | 5.4 | 182.3 | -62.2 |
| 3-Hydroxybutyric Acid | 0.0025 | 0.002 | - | 16.2 | 597.3 | - |
| Oleic Acid | 0.0035 | 0.005 | 0.035 | 5.5 | 185.0 | -49.9 |
| Cholesterol | 0.0044 | - | 0.006 | 9.6 | - | 76.3 |
| L-5-Oxoproline | 0.0046 | 0.010 | 0.025 | 17.8 | 73.7 | -44.0 |
| 2-Keto-3-Methylvaleric Acid | 0.0062 | 0.023 | 0.017 | 10.3 | 83.6 | -48.9 |
| Myo-Inositol | 0.0071 | 0.005 | - | 18.5 | 90.9 | - |
| Glycolic Acid | 0.0095 | - | 0.008 | 19.8 | - | -50.0 |
| Palmitic Acid | 0.0103 | 0.015 | - | 4.5 | 200.0 | - |
| 13-Octadecenoic Acid | 0.0116 | 0.016 | - | 5.7 | 118.6 | - |
| Palmitoleic Acid | 0.0141 | 0.014 | - | 5.2 | 186.5 | - |
| Unknown 11 | 0.0175 | 0.019 | - | 15.8 | -74.3 | - |
| L-Hydroxyproline | 0.0248 | - | 0.020 | 15.4 | - | -66.2 |
| Hippuric Acid | 0.0302 | 0.029 | - | 11.0 | 749.9 | - |
| Glycine | 0.0550 | - | 0.049 | 16.9 | - | -59.4 |

CCC – chronic chagasic cardiomyopathy; CTRL – control; IDC – idiopathic dilated cardiomyopathy; QC – quality control; RSD – relative standard deviation.
